# Isotopic Overlap of Invasive and Native Consumers in the Food Web of Lake Trasimeno (Central Italy)

**DOI:** 10.3390/biology12091270

**Published:** 2023-09-21

**Authors:** Davide Cicala, Maria Teresa Guerra, Roberta Bardelli, Cristina Di Muri, Alessandro Ludovisi, Salvatrice Vizzini, Giorgio Mancinelli

**Affiliations:** 1 Department of Biology, University of Rome Tor Vergata, Via della Ricerca Scientifica 1, 00133 Rome, Italy; ccldvd01@uniroma2.it; 2 Department of Biological and Environmental Sciences and Technologies—DiSTeBA, University of Salento, SP Lecce-Monteroni, 73100 Lecce, Italy; mariateresa.guerra@unisalento.it; 3 Department of Earth and Marine Sciences—DiSTeM, University of Palermo, Via Archirafi 22, 90133 Palermo, Italy; roberta.bardelli@unipa.it (R.B.); salvatrice.vizzini@unipa.it (S.V.); 4 Italian National Research Council, Institute of Research on Terrestrial Ecosystems—CNR-IRET, University of Salento, SP Lecce-Monteroni, 73100 Lecce, Italy; cristina.dimuri@cnr.it; 5 Department of Chemistry, Biology and Biotechnologies, University of Perugia, Via Elce di Sotto 8, 06123 Perugia, Italy; alessandro.ludovisi@unipg.it; 6 Consorzio Nazionale Interuniversitario per le Scienze del Mare—CoNISMa, Piazzale Flaminio 9, 00196 Rome, Italy; 7 Italian National Research Council, Institute of Marine Biological Resources and Biotechnologies—CNR-IRBIM, Via Pola 4, 71010 Lesina, Italy

**Keywords:** invasive species, non-indigenous species, ecological impact, δ^13^C and δ^15^N, isotopic niche, *Procambarus clarkii*

## Abstract

**Simple Summary:**

An in-depth study of the feeding habits characterizing bioinvaders may provide key information on the magnitude of their impacts on recipient communities. Specifically, if invaders’ trophic niche is superimposed on that of native species, interspecific competition may increase, resulting in negative consequences for the competing species; alternatively, trophic niche divergence may occur, facilitating the invaders’ integration into the community. In the present study, the analysis of carbon and nitrogen stable isotopes was used to investigate the trophic overlap of native and non-indigenous consumers. We found a generally low degree of isotopic overlap in both the invertebrate and fish assemblage, a condition that may facilitate coexistence and, in turn, limit the strength of invaders’ impact. The only exception was the Louisiana crayfish *Procambarus clarkii*, which was demonstrated to interact with a wide spectrum of native invertebrate species, confirming the necessity of guaranteeing appropriate measures of control and mitigation of its ecological impacts.

**Abstract:**

An advanced characterization of the trophic niche of non-indigenous species (NIS) may provide useful information on their ecological impact on invaded communities. Here, we used carbon and nitrogen stable isotopes to estimate pairwise niche overlaps between non-indigenous and native consumers in the winter food web of Lake Trasimeno (central Italy). Overall, a relatively low pairwise overlap of isotopic niches was observed between NIS and native species. The only exception was the Louisiana crayfish *Procambarus clarkii*, which showed a relatively high and diffuse overlap with other native invertebrates. Our findings highlighted a high niche divergence between non-indigenous and native species in Lake Trasimeno, suggesting a potentially low degree of interspecific competition that may facilitate coexistence and, in turn, limit the strength of impacts. The divergent results obtained for the Louisiana crayfish indicate that additional control measures for this invasive species are needed to mitigate its impact on the Lake Trasimeno system.

## 1. Introduction

The introduction and establishment of non-indigenous species (NIS hereafter) represents one of the most important anthropogenic threats to the biodiversity, functioning, and integrity of freshwater ecosystems [1,2]. This is particularly evident when NIS establish themselves and become invasive, altering the structure and functions of recipient communities and whole ecosystems and ultimately causing environmental and economic harm [3,4,5,6].

A growing body of evidence is accumulating on the negative impacts of bioinvaders in both lentic and lotic environments, but an overwhelming majority of these investigations are focused on the effects of single species or single taxonomic groups [7]; see also the studies used for the meta-analyses performed by, e.g., [8,9,10,11]. In freshwater environments, however, repeated introductions may take place, resulting in a generalized occurrence of multispecific NIS assemblages [12,13]. Multiple invaders are recognized to exert a wide spectrum of cumulative effects, from synergistic to negligible to antagonistic [14,15,16,17]; yet, compared with plant species, multiple animal invasions have generally received less attention, in particular in freshwater ecosystems [18]. The establishment of invasive consumers necessarily implies a “rewiring” of trophic interactions directly through predation or indirectly through competition [19,20,21,22,23]. From this perspective, the study of interspecific feeding interactions at a whole-food-web scale may provide important insights into the combined ecological effects and impacts of multiple invaders on native species.

In the present study, we focused on Lake Trasimeno, a laminar basin in central Italy characterized by a diverse assemblage of non-indigenous species of fish and invertebrates [24]. The investigation was performed in winter with the aim of estimating the trophic overlap between NIS and native species and to assess their potential for competition. If trophic resources are limiting, NIS establishment within a recipient community can generally result in increased interspecific competition; this may ultimately be reflected in competitive exclusion and local extinction of the inferior competitor or, alternatively, in trophic niche divergence, which would facilitate the integration of invaders and their coexistence with indigenous species [25,26,27]. Here, we conjectured a generally high degree of trophic overlap and potential for competition between native and non-indigenous consumers, given the occurrence of highly invasive species in both the invertebrate and fish assemblages of the lake (see further in the next sections). However, in temperate lakes, the abundances of primary producers, intermediate consumers, and top predators undergo strong seasonal fluctuations with minima generally observed in winter; see, e.g., [28,29,30] for Lake Trasimeno. Accordingly, an alternative hypothesis was that the low abundances and metabolic requirements of native and non-indigenous consumers in the colder season should correspond to conditions of weak competitive interactions and reduced trophic overlap [31,32]. We tested these hypotheses using stable isotope analysis (SIA). This methodology has in the last decades gained huge popularity in the study of aquatic food webs and the assessment of the response of marine and freshwater ecosystems to anthropogenic pressures, including bioinvasions [33,34]. In recent years, methods for estimating trophic niche space—conventionally relying on direct observations and stomach content analysis—have improved by integrating SIA-based approaches (“isotopic niche” in [35,36]). The δ^13^C and δ^15^N values of living organisms vary at both an inter- and intraspecific level; accordingly, consumers will occupy a different isotopic space depending on the resources they exploit, making an organism’s isotopic niche a useful proxy of its trophic niche [37,38]. Carbon and nitrogen stable isotopes were measured in winter in fish, invertebrates, and basal resources collected from a littoral site of the lake. We generated Bayesian standard ellipse areas of each species representing relative niche widths in the isotopic space, and pairwise overlaps were computed. In addition, the mean proportional overlap between the native and non-indigenous assemblages (all species combined) was used as a metric for estimating the cumulative influence of the latter on the transfer of energy and matter in the lacustrine food web [39,40,41].

## 2. Materials and Methods

### 2.1. Site Description

**Figure 1:**
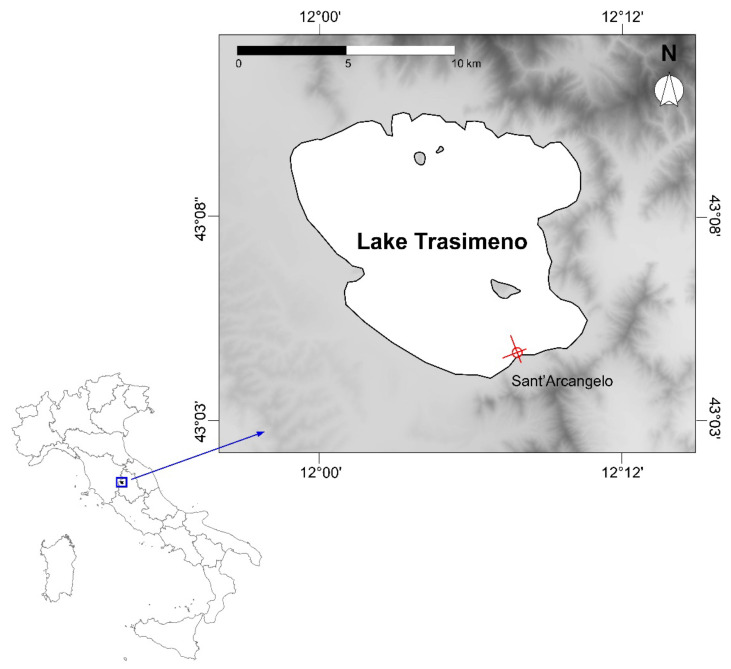
Lake Trasimeno. The study location is highlighted in red.

This study was performed in Lake Trasimeno (43.133283° N, 12.100064° E, central Italy; Figure 1). The basin is the largest laminar lake in Italy (124 km^2^, average depth: 4.7 m, maximum depth: 6.3 m). It is located 257 m above sea level, it has a single artificial effluent, and it is fed by several ephemeral creeks. Given the relatively small extension of the watershed (396 km^2^), its hydrological regime is driven by precipitation, and strong seasonal and interannual oscillations in water level and chemistry are observed [42,43]. Further details on the lake’s morphometric and hydrological characteristics are provided in Ludovisi and Gaino [42] and in Bresciani et al. [44].

The lake is included in a regional natural park within the Natura 2000 European network. The littoral zones are generally muddy and dominated by common reeds (*Phragmites australis* (Cav.) Trin. ex Steud.), with dense beds of aquatic macrophytes of the genera *Myriophyllum*, *Stuckenia*, and *Vallisneria* extending in summer especially along the southern coasts of the lake [45]. The native macroinvertebrate community includes a diverse assemblage of annelid, mollusk, insect, and crustacean taxa [46]. Conversely, the assemblage of native fish species currently comprises only five species, i.e., *Esox cisalpinus* Bianco & Delmastro 2011, *Anguilla anguilla* Linneus 1758, *Tinca tinca* Linnaeus 1758, *Scardinius erythrophthalmus* Linnaeus, 1758, and *Squalius squalus* Bonaparte 1837. Two additional species, *Sarmarutilus rubilio* Bonaparte 1837 and *Cobitis bilineata* Canestrini 1865, are considered to be extinct since the 1970s [46,47].

Phytoplankton are characterized by wide seasonal fluctuations and are dominated in summer by the cyanophyceans *Cylindrospermopsis* spp., whereas in winter, chlorophyceans of the genera *Hyaloraphidium* and *Scenedesmus* together with cyanophyceans of the genus *Leptolyngbya* represent the most abundant taxa [48,49]. Similarly, remarkable seasonal variations characterize the abundance and composition of the zooplankton, dominated in summer by the cladoceran *Daphnia galeata* Sars 1864 and by copepods of the genus *Cyclops* in winter [48,50,51]. During the last century, the lacustrine community has been drastically altered by the introduction of several NIS of different origins (Table 1). They comprise invertebrates (e.g., the decapods *Procambarus clarkii* and *Dikerogammarus villosus*, the bivalves *Dreissena polymorpha* and *Sinanodonta woodiana*, and the tubificid *Branchiura sowerbyi*) and 15 species of fish, including *Ameiurus melas*, *Carassius auratus*, *Lepomis gibbosus*, *Micropterus salmoides*, and *Perca fluviatilis* [24,47,52].

### 2.2. Sample Collection and Laboratory Procedures

In early February 2018, fish and crayfish were captured by fishers operating fyke nets in Sant’Arcangelo, a locality in the southern sector of the lake (43.089788° N, 12.156246° E). Nets were located at an approximate distance of 50 m from the coast at a depth of 2–2.5 m. Collected specimens were transferred to the laboratory in refrigerated containers (4 °C), where they were euthanized by thermal shock (−80 °C for 10 min), identified to the lowest taxonomic level possible, and enumerated. Subsequently, a ruler was used to determine to the nearest mm the total standard length of fish specimens (i.e., from the tip of the snout to the posterior end of the last vertebra); a caliper was used to measure in mm the total length of crustacean individuals from the tip of the rostrum to the end of the telson.

For stable isotope analysis, the dorsal muscle tissue of fish and the caudal muscle of crustaceans was dissected from each specimen using a ceramic scalpel, dried (60 °C, >1 week), and powdered using a mortar and a pestle. Subsequently, subsamples (0.8 ± 0.02 mg, mean ± 1 SE) were pressed into Ultra-Pure tin capsules (Costech Analytical Technologies, Valencia, CA, USA) and analyzed using an elemental analyzer connected to an isotope ratio mass spectrometer (Thermo Scientific Flash EA 1112, Waltham, MA, USA and Delta Plus XP, Suzhou, China, respectively). Isotopic contents were expressed in conventional δ unit notation as ‰ deviations from international standards:

(1) $$\delta^{13}C\;or\;\delta^{15}N=\left[\left(R_{sample}/R_{standard}\right)-1\right]\times 1000$$

where R = ^13^C/^12^C or ^15^N/^14^N. Pee Dee belemnite (PDB) limestone carbonate and atmospheric nitrogen (N_2_) were used as standards for carbon and nitrogen isotope ratios, respectively. Analytical precision based on the standard deviation of replicates of internal standards (International Atomic Energy Agency IAEA-NO-3 for δ^15^N and IAEA-CH-6 for δ^13^C) was 0.2‰ for both δ^15^N and δ^13^C.

Invertebrates were collected in a shallow embayment (approximate depth = 1 m) situated in front of the area where fish and crayfish were sampled. Details of the location are provided in Mancinelli et al. [57], while information on the sampling procedures are included in Mancini et al. [58] and Ludovisi et al. [59]. In brief, the embayment has muddy bottoms and artificial rocky shores, characterized in winter by accumulations of decaying plant material, including *P. australis* leaf litter and mixed detritus of macrophytes belonging to the genera *Myriophyllum*, *Potamogeton*, and *Vallisneria*. At the time of the sampling, in the embayment, the temperature and dissolved oxygen concentration of surficial water (depth = 10–12 cm) were 12.3 ± 0.2 °C and 9.8 ± 0.8 mg L^−1^, respectively (mean ± 1 SE, *n* = 3; YSI^®^ 556 MPS, Yellow Springs, OH, USA). A handheld pond net (mesh size = 1 mm) was swept five times in floating leaf litter accumulations to capture macroinvertebrates; additional specimens were collected by hand from rocks and artificial hard substrata. Samples were subsequently placed in Falcon tubes or in other plastic buckets containing filtered lake water. Samples of the superficial sediment layer (3 replicates) were collected using a methacrylate core (400 mm length, 114 mm ∅) driven into the sediment to a depth of approximately 10 cm and were then transferred to plastic bags.

All samples were transported in refrigerated containers (4 °C) to the laboratory, where invertebrates were identified to the lowest taxonomic level, enumerated, kept in distilled water for 12 h to clear gut contents, and eventually euthanized by thermal shock (−80 °C for 10 min). The total length of each individual was determined to the nearest 0.1 mm either using a digital caliper for palaemonids (see Results (Section 3)) or a stereo microscope (Nikon^®^ SMZ1270, Tokyo, Japan) equipped with a CCD camera for the remaining taxa. For gastropods, the maximum shell length was measured. Subsequently, the caudal muscle of each palaemonid was dissected and dried (60 °C, >1 week); all the remaining invertebrates were dried whole with the exclusion of gastropods, which had their shells removed. Sediment samples were wet-sieved on a 1 mm screen; invertebrates retained in the sieve were collected and processed according to the procedures already described.

The isotopic data obtained in the present investigation were complemented with those published in Mancini et al. [58] and Ludovisi et al. [59]. They were performed at the same location as this study and include δ^13^C and δ^15^N values of benthic invertebrates and zooplankton. The study by Ludovisi et al. [59] was carried out in 2018 and coincided with the present investigation, while that of Mancini et al. [58] was performed in February 2016. The isotopic characteristics of the components of the food web at the study site were assumed to have remained unchanged between the sampling years. The assumption was tested for a subset of invertebrate species (Appendix A; see the next section).

### 2.3. Data Analysis

In general, all statistical analyses were carried out in the R statistical environment v. 4.2.2 [60]. For univariate analyses, data were checked for normality and homoscedasticity (Shapiro–Wilks and Levene’s tests, respectively) and log- or square root-transformed if required. When significant effects were detected by ANOVA tests, post hoc bivariate comparisons were performed using Tukey HSD tests. Bivariate relationships were tested using Pearson’s coefficient of correlation, while F-tests were used to verify differences between slopes or intercepts.

To preliminarily verify whether the invertebrate taxa sampled in 2016 and 2018 (Appendix A) differed significantly between years in their isotopic values and CN contents, a Euclidean distance similarity matrix of Z-scaled δ^13^C, δ^15^N, and total carbon and nitrogen (both expressed as %) values was calculated. Subsequently, a type III (partial) permutational analysis of variance [61] (PERMANOVA hereafter) with 9999 permutations was performed using the function adonis in the vegan package v. 2.6-2 [62] with “species” and “year” as the fixed and random factors, respectively. Since no significant effects were detected (see Results), the two isotopic datasets were cumulated and treated in further analyses as one.

Independently from the sampling date, the tissues of several invertebrates and fish showed C:N ratios > 3.5 (Appendix A), indicating a significant contribution of lipids to the tissues’ carbon pool [63]. Since lipids are depleted in ^13^C compared to proteins and carbohydrates and can significantly bias δ^13^C estimations [64], samples with a C:N ratio > 3.5 had their δ^13^C values mathematically corrected for lipid content [63].

Isotopic niche overlaps for non-indigenous and native consumers were estimated using the SIBER package v. 2.1.6 [65]; consumers’ standard ellipse area (SEA, expressed in ‰^2^) was used as a measure of the core population isotopic niche [66]. Given the different number of specimens per taxon included in the analyses (Appendix A), we calculated a sample-size-corrected version (SEA_c_ hereafter) [66] representing the core (40%) isotopic niche area and allowing for robust comparisons across species of varying sample sizes. SEA_c_ estimations were used for illustrative purposes; for interspecific statistical comparisons, we calculated the Bayesian equivalent SEA_B_ of SEA_c_ [66] using 100,000 posterior iterations of SEA_c_ to compute credible intervals. Specifically, pairwise contrasts were performed by calculating the probability that the SEA_B_ of one species was different from that of a second one with a probability of at least 95% [66].

SEA_c_ estimations were further used to calculate interspecific isotopic niche overlaps. They were determined assuming negligible competitive interactions between invertebrate and fish consumers; in addition, for each of the two groups, overlaps were calculated between NIS and native species as well within NIS and native assemblages separately.

Overlaps between two species were expressed as the % ratio of the estimated overlap itself and the sum of the nonoverlapping area of the ellipses for each species [66], a measure conceptually consistent with other classical symmetric indices, such as Pianka’s niche overlap index O [67]. Overlaps were considered significant when the shared isotopic space between species was >60%, a criterion identical to that used by Schoener for his dietary overlap index [68].

## 3. Results

### 3.1. Invertebrates

**Figure 2:**
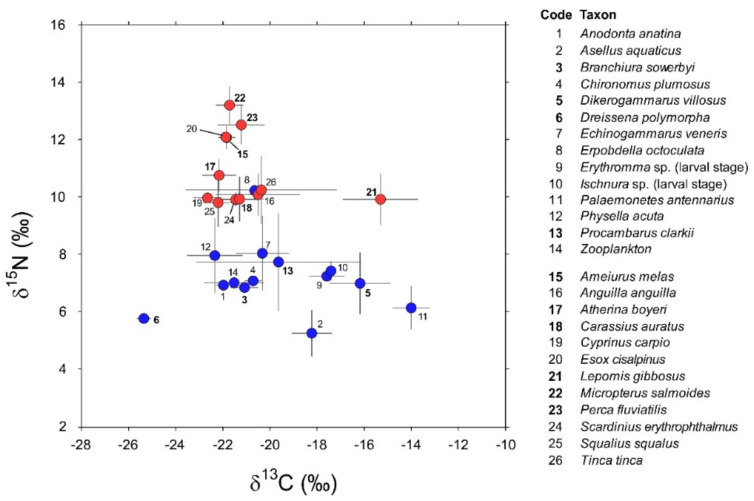
δ^13^C and δ^15^N values (Mean ± 1 SD) of consumers (invertebrates: codes 1–14; fish: codes 15–26; blue and red circles, respectively) characterizing the food web of Lake Trasimeno in winter. Non-indigenous species are indicated in bold.

Invertebrate taxa sampled in 2016 and 2018 (Appendix A) showed negligible between-year variations in their isotopic values (PERMANOVA, factor “year”: Pseudo-F_1,72_ = 0.79, P_Monte Carlo_ = 0.44; interaction factor “species × year”: Pseudo-F_5,61_ = 0.3, P_Monte Carlo_ = 0.97). Additionally, post hoc taxon-specific comparisons highlighted negligible temporal differences (min *t* = 1.16, P_Monte Carlo_ = 0.27 for *P. clarkii*). Accordingly, the data collected during the two sampling occasions were cumulated and treated as a single dataset containing isotopic measurements for a total of 163 specimens belonging to 14 invertebrate taxa (Appendix A). Their mean δ^13^C values varied considerably between −25.6 in the zebra mussel *D. polymorpha* and −14.1 in the pond shrimp *P. antennarius* (Figure 2).

The isopod *Asellus aquaticus* and the leech *Erpobdella octoculata* were the most depleted and enriched in ^15^N, respectively (Figure 2; 5.2 ± 0.8 vs. 10.2 ± 0.2, mean ± 1 SD). Overall, invertebrates showed significant interspecific differences in isotopic composition (PERMANOVA, Pseudo-F_13,162_ = 26.3, P_Monte Carlo_ = 0.0001); this result was generally confirmed by further post hoc bivariate comparisons (Table A2 in Appendix B). Noticeable exceptions were *Chironomus plumosus* vs. *Branchiura sowerbyi* and *Echinogammarus veneris* and, most importantly, *Procambarus clarkii* vs. a taxonomically heterogeneous assemblage including most of the invertebrates with the exclusion of *Dikerogammarus villosus*, *Dreissena polymorpha*, and *Physella acuta* (Table A2 in Appendix B).

**Figure 3:**
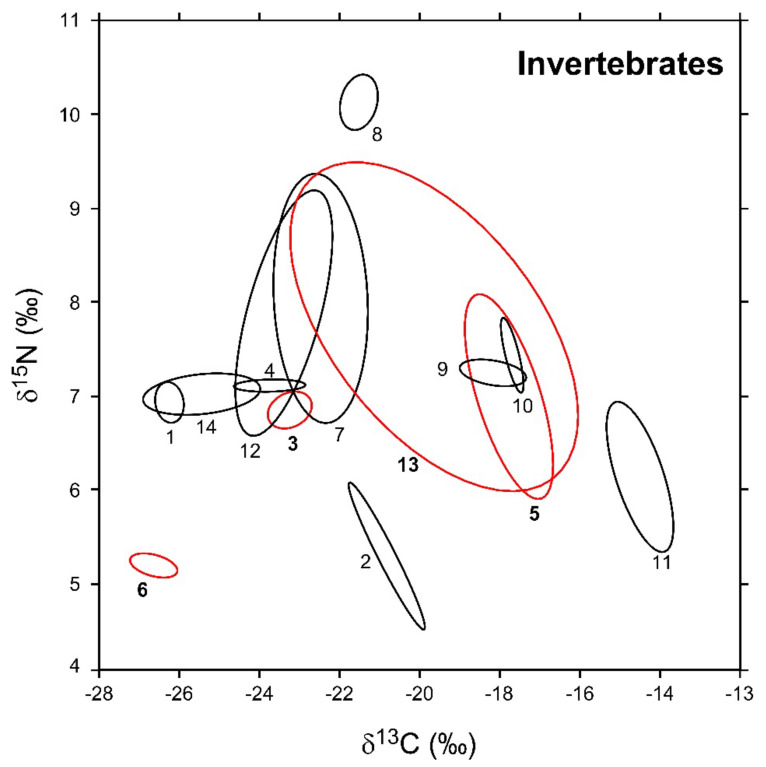
Sample-size-corrected Bayesian standard ellipse areas (SEAc) calculated from δ^13^C and δ^15^N values of invertebrate consumers. Black and red ellipses refer to native and non-indigenous taxa, respectively; see Figure 2 for the corresponding identification codes.

In Figure 3, the sample-size-corrected standard ellipse areas (SEA_c_) of invertebrate consumers are illustrated, while numerical values are reported in Table 2 together with the respective Bayesian estimates (SEA_B_). Overall, modal SEA_B_ and SEA_c_ values were in good agreement, and the latter always fell within SEA_B_ 95% confidence intervals. The robustness of Bayesian estimations against potential biases related to variations in sample size was corroborated by the negligible relationship observed between SEA_B_ values and the number of specimens analyzed for each taxa (Pearson *r* = 0.27, *p* = 0.35, d.f. = 12).

Isotopic niche areas varied across the different taxa by a factor > 100 (Table 2). Among NIS, *P. clarkii* showed the highest SEA_B_ value (16.9‰^2^), one order of magnitude larger than *D. villosus* (2.8‰^2^). *B. sowerbyi* and *D. polymorpha*, conversely, were characterized by the lowest SEA_B_ estimations (0.3 and 0.2‰^2^, respectively). Bivariate comparisons indicated significant differences in isotopic niche areas among all the taxa with the exclusion of *B. sowerbyi* and *D. polymorpha* (probability tests, *B. sowerbyi* ≠ *D. polymorpha*: *p* = 0.48; *p* > 0.95 for all the remaining comparisons). Similar to what was observed for NIS, native invertebrates showed a high heterogeneity in their modal SEA_B_ estimations (Table 2); *P. acuta* showed the largest value, close to that of *E. veneris* (4.7 and 4.5‰^2^, respectively; probability test *p* = 0.62). In turn, they were significantly different from SEA_B_ values determined for both *P. clarkii* and *D. villosus* (probability tests, *p* always > 0.95). In contrast, *Ischnura* sp. and *C. plumosus* showed the smallest SEA_B_ (0.1‰^2^). *Erythromma* sp., *A. anatina*, and *E. octoculata* showed areas ranging between 0.3 and 0.2‰^2^ (*p* < 0.95 for all bivariate comparisons), whereas for a second taxonomically heterogeneous group including *P. antennarius*, zooplanktonic crustaceans, and *A. aquaticus*, the estimated areas ranged between 1.6 and 1‰^2^ (*p* < 0.95 for all bivariate comparisons).

**Figure 4:**
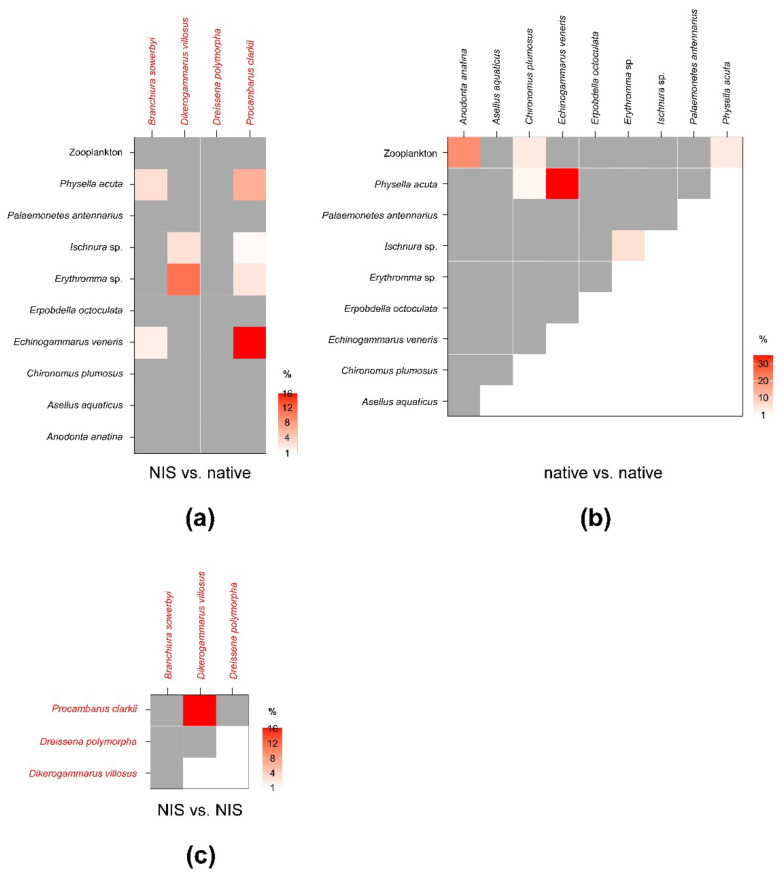
Percent isotopic niche overlaps of Bayesian standard ellipse areas (SEA_B_) of non-indigenous vs. native (**a**), native vs. native (**b**), and non-indigenous vs. non-indigenous invertebrate consumers (**c**). Heat maps were built using modal overlaps; the reader should refer to Table A4, Table A5 and Table A6 in Appendix C for 95% confidence intervals of modal values and the corresponding SEA_c_ estimations. Please note the different % scales.

SEA_c_ and SEA_B_ percent overlaps between NIS and native invertebrate species were generally low and well below the critical limit of 60% (Figure 4; see Table A4 in Appendix C for modal SEA_B_ values and the respective 95% confidence intervals). The mean of nonzero SEA_c_ and SEA_B_ % overlaps was 3.3% and 3.4%, respectively, with values ranging between a minimum approximating 0% for *P. clarkii* vs. *A. aquaticus* and a maximum of 16 (SEA_c_)–18% (SEA_B_) for *P. clarkii* vs. *E. veneris*. Noticeably, the mean % overlap among native species was 7.1% and 6.4% (SEA_c_ and SEA_B_ % overlaps, respectively), with values ranging between a minimum approximating 0% for *E. veneris* vs. *A. aquaticus* and a maximum of 35.2 (SEA_c_)–30.8% (SEA_B_) for *E. veneris* vs. *P. acuta* (Figure 4; Table A5 in Appendix C).

Beside *E. veneris*, *P. clarkii* showed overlaps > 1% also with *P. acuta* (8.4%) and with *Erythromma* sp. (1.8%), while *D. villosus* overlapped with dragonfly nymphs (i.e., 9.4% with *Ischnura* sp. and 2.9% with *Erythromma* sp.). Other NIS showed negligible overlaps with native taxa; however, *P. clarkii* showed a relatively high SEA_B_ % overlap with *D. villosus* of 15.8% (8.4–21.3%, 95% CI; Figure 4; Table A6 in Appendix C), indicating a potential interaction.

### 3.2. Fish

Isotopic analyses were performed on a total of 140 specimens belonging to 12 fish taxa (Table A1, Appendix A). In general, they showed significant interspecific differences in isotopic composition (PERMANOVA, Pseudo-F_11,139_ = 50.9, P_Monte Carlo_ = 0.001). The highest δ^15^N values were observed in *Micropterus salmoides* together with *Perca fluviatilis*, *Esox cisalpinus*, and *Ameiurus melas*, the latter two species showing negligible isotopic differences (Table A3, Appendix B). The remaining species clustered in a group characterized by a lower enrichment in ^15^N and generally showing significant interspecific variations, with some notable exceptions represented by *Carassius auratus* vs. *Cyprinus carpio*, *Scardinius erythrophthalmus*, and *Anguilla anguilla*, the latter two characterized by negligible isotopic differences (Table A3, Appendix B). *Lepomis gibbosus* had δ^15^N values consistent with those characterizing this second group but with δ^13^C values significantly more enriched (Figure 2 and Table A3, Appendix B).

**Figure 5:**
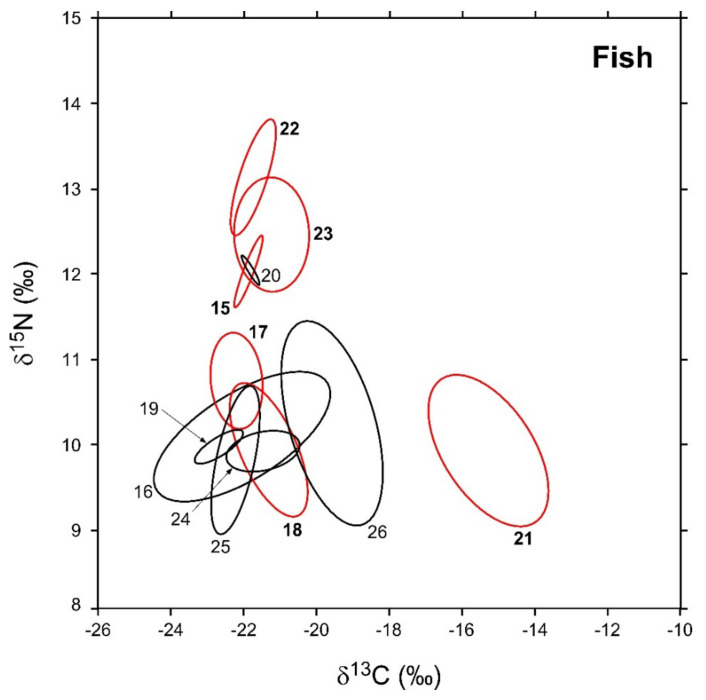
Sample-size-corrected Bayesian standard ellipse areas (SEAc) calculated from δ^13^C and δ^15^N values of fish consumers. Black and red ellipses refer to native and non-indigenous taxa, respectively; see Figure 2 for the corresponding identification codes.

In Figure 5, the sample-size-corrected standard ellipse areas (SEA_c_) of invertebrate consumers are illustrated, while numerical values are reported in Table 3 together with the respective Bayesian estimates (SEA_B_). As observed for invertebrates, modal SEA_B_ and SEA_c_ estimations were in good agreement, and the latter were always included within SEA_B_ 95% confidence intervals. The robustness of Bayesian estimations against potential biases related with variations in sample size was confirmed by the negligible relationship observed between SEA_B_ values and the number of specimens analyzed for each taxa (Pearson *r* = 0.38, *p* = 0.21, d.f. = 10).

In general, SEA_B_ values varied across the different taxa by one order of magnitude (Table 3). Among NIS, *L. gibbosus* and *A. melas* were characterized by the largest and smallest areas (3.8 and 0.3‰^2^, respectively), while *P. fluviatilis*, *C. auratus*, *A. boyeri*, and *M. salmoides* showed intermediate values ranging between 2 and 0.8‰^2^. Bivariate comparisons indicated significant differences with a probability > 95% in the SEA_B_ of all the species with the exception of *P. fluviatilis* vs. *C. auratus* and *A. boyeri* vs. *M. salmoides* (probability tests, max *p* = 0.73 for the comparison *P. fluviatilis* ≠ *C. auratus*). Native fish varied in SEA_B_ values by a factor > 100, ranging between maxima of 4.2 and 3.8‰^2^ observed for *Tinca tinca* and *A. anguilla*, respectively, and a minimum of 0.02‰^2^ characterizing *E. cisalpinus*. *S. squalus*, *S. erythrophthalmus*, and *C. carpio* showed intermediate values ranging between 1.4 and 0.2‰^2^; all fish taxa showed significant interspecific differences in SEA_B_ values with the exception of *T. tinca* and *A. anguilla* (probability test, *p* = 0.56).

**Figure 6:**
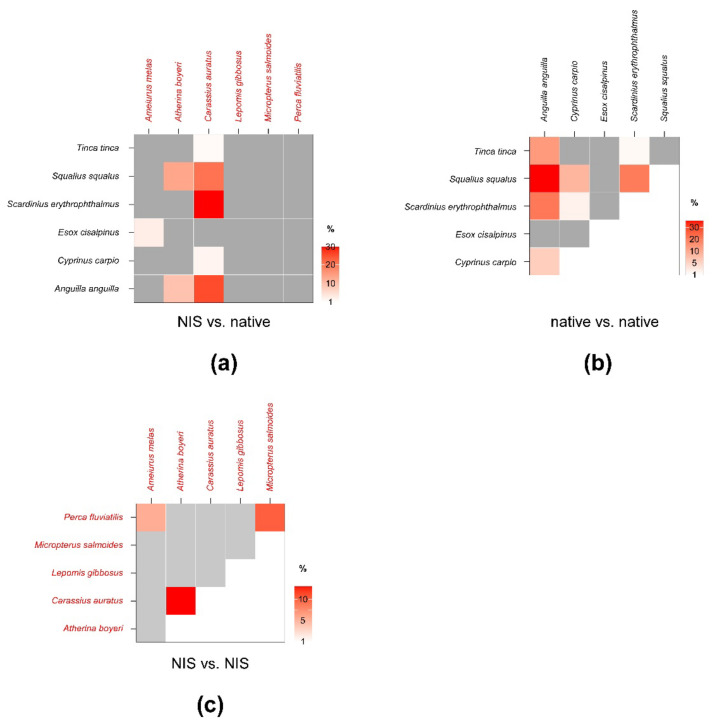
Percent isotopic niche overlaps of Bayesian standard ellipse areas (SEA_B_) of non-indigenous vs. native (**a**), native vs. native (**b**), and non-indigenous vs. non-indigenous fish consumers (**c**). Heat maps were built using modal overlaps; the reader should refer to Table A7, Table A8 and Table A9 in Appendix D for 95% confidence intervals of modal values and the corresponding SEA_c_ estimations. Please note the different % scales.

SEA_c_ and SEA_B_ percent overlaps between NIS and native fish species were generally higher than those observed for invertebrates (Figure 6, see Table A7 in Appendix D for modal SEA_B_ values and the respective 95% confidence intervals), yet they were always below the 60% threshold. The mean of nonzero SEA_c_ and SEA_B_ % overlaps was 3.8% and 3.6%, respectively, with values ranging between a minimum approximating 0% for *M. salmoides* vs. *E. cisalpinus* and a maximum of 25.1 (SEA_B_)–29% (SEA_c_) for *C. auratus* vs. *S. erythrophthalmus.* Similar to what was observed for invertebrates, the mean % overlap among native species was higher than that determined between NIS and native taxa, i.e., 7.5% and 6.2% (SEA_c_ and SEA_B_ % overlaps, respectively), with values ranging between a minimum approximating 0% for *E. cisalpinus* vs. S. *erythrophthalmus*, *S. squalus*, and *A. anguilla* and a maximum of 18.1 (SEA_B_)–22.9% (SEA_c_) for *A. anguilla* vs. *S. squalus* (Figure 6; Table A8 in Appendix D). Noticeably, the mean of nonzero SEA_c_ and SEA_B_ % overlaps among NIS was 2.9% and 3.7%, respectively, with values ranging between a minimum approximating 0% for *A. melas* vs. *C. auratus* and *A. boyeri* and a maximum of 13.1 (SEA_c_)–18.8% (SEA_B_) for *C. auratus* vs. *A. boyeri* (Figure 6; Table A9 in Appendix D).

## 4. Discussion

Of the two contrasting hypotheses originally formulated in this study, only the second received support from the data, as the results indicated that a low potential trophic overlap occurs between non-indigenous and native consumers in the winter food web of Lake Trasimeno. The average percent overlap, measured using either conventional sample-size-corrected standard ellipse areas (SEAc) or Bayesian standard ellipse areas (SEA_B_), was below 4% for both the invertebrate and the fish assemblage. Additionally, pairwise % overlaps estimated between NIS and native species were below 16% for invertebrates and 30% for fish, far from the 60% critical threshold generally acknowledged to be related to active competitive interactions, e.g., [68,69]. The highest overlaps were observed among invertebrates for the Louisiana crayfish *P. clarkii* and to a minor extent for the killer shrimp *D. villosus*, while the goldfish *C. auratus* was characterized by the highest and most diffuse overlaps with native fish species. These findings have general theoretical implications but deserve to be discussed while focusing also on a species-specific perspective.

### 4.1. General Considerations

The low overlap indices observed between NIS and native consumers indicates that low potential competition occurs and that a stable coexistence equilibrium takes place in both the invertebrate and fish assemblages. In addition, it suggests that NIS play important functional roles in the flux of energy and matter from basal to higher trophic levels and have become pivotal in supporting the whole food web of Lake Trasimeno.

Mutual coevolution shapes competitors’ niches in natural communities [70]; accordingly, coexisting species should exhibit relatively low overlap in resource utilization; alternatively, if competition for limited resources is ongoing, a high niche overlap should occur [70,71,72]. In the context of bioinvasions, this latter scenario generally characterizes non-indigenous populations in their post-introduction and early-establishment phases, generally coupled with anomalously high abundances, e.g., [73]. The duration of coexistence with native species in the recipient food web is considered a key factor driving relevant ecological and evolutionary processes [74,75], as short-term coexistence between NIS and native species with a similar trophic ecology may induce high niche overlaps [76,77]. In contrast, long-term coexistence (>10 years) has been shown to be paralleled by shifts in diet and habitat segregations with important consequences for competitors’ trophic interactions [78,79,80,81]. Here, no significant relationships were observed in, e.g., the time since the introduction of NIS in Lake Trasimeno and their mean overlaps with native species (Pearson *r* = −0.21, *p* = 0.59, d.f. = 8). Indeed, for invertebrates as well as for fish, the observed low overlaps can be partially explained by differences in trophic habits; however, even focusing on species belonging to the same trophic guild, such as filter-feeding bivalves (*D. polymorpha* vs. *A. anatina*), detritivorous amphipods and isopods (*D. villosus* vs. *E. veneris* and *A. aquaticus*), or predatory fish (*M. salmoides* vs. *E. cisalpinus*), negligible or very limited overlaps have been observed, with larger overlaps determined within native- or NIS-only assemblages. The relatively long invasion history experienced by the Lake Trasimeno community can be hypothesized as the main determinant of the limited isotopic overlaps observed here. Among invertebrates, *D. villosus* can be considered an exception, as it appeared in 2017, whereas the remaining species were introduced around 2000 or earlier (Table 1); among fish, the most recent introductions were *C. auratus* and *M. salmoides* in 1990. Thus, it is plausible that the low overlap observed in the isotopic space may result from the peculiar conditions of relatively low abundance and metabolic requirements of both native and non-indigenous consumers taking place in Lake Trasimeno during the cold season as well as from long-term phenomena of adaptation and partitioning of the available resources in order to reduce competition and promote coexistence. Other factors related with, e.g., life history and geographic origin of NIS, cannot be excluded, as they have been indicated to play a role in freshwater communities characterized by multiple invasions [82].

Interestingly, the low overlap between NIS and native consumers suggests a high diversification in the contribution of both invertebrate and fish species to the channelling of matter and energy from basal resources to higher trophic levels. In other words, NIS appear to have acquired in the years after their introduction and establishement a structural as well as a functional role in the food web of Lake Trasimeno. For island ecosystems, the eradication of invasive species has been indicated to exert unquestionable benefits to the extant native biota, but empirical observations have also shown that these benefits can vary widely and unpredictably, and that adverse consequences may take place due to the disruption of the novel functional relationships generated by the invaders’ “surprise effects” in [83,84,85,86]. Thus, for Lake Trasimeno, this may have important implications from a management perspective: with the exclusion of *P. clarkii* and *C. auratus*, both showing low but diffuse overlaps with native species, any mitigation strategy based, e.g., on the reduction in the abundance of a NIS should also take into consideration a variation in the functioning of the food web.

### 4.2. Species-Specific Issues

*Procambarus clarkii* was shown to have the largest nichecluding among the invertebrate species included in this study, confirming the results of other isotopic investigations performed in both lentic and lotic environments [82,87]. Current information on the trophic ecology of this omnivorous species mostly pertains to food selection and dietary overlap with other crayfish species [88,89,90,91]; only recently, Wu and colleagues [92] verified in a Chinese reservoir a substantial resource overlap between *P. clarkii* and native crustaceans and gastropods, indicating the potential for the crayfish to exert negative impacts through competition. Here, *P. clarkii* overlapped with a number of native species, including the amphipod *E. veneris* and the gastropod *P. acuta*. Further comparative studies on the trophic niche of the crayfish are needed, specifically addressing potential competitive interactions with representatives of the detritivorus guild, native as well non-indigenous; here, *P. clarkii* showed a relatively high overlap also with the killer shrimp *D. villosus*. Indeed, while investigations based on conventional gut content analysis suggest similarity in dietary habits [93,94], the results of the only isotopic study including these two species, does not lend support to this view [82]; thus, to date, the nature and strength of the interaction between *P. clarkii* and *D. villosus* remain virtually unexplored.

Among fish taxa, the pumpkinseed *Lepomis gibbosus* was characterized by a large niche, yet no overlaps occurred with other fish species, either native or non-indigenous. The species was characterized by δ^13^C values far more enriched than those characterizing other fish species (Figure 2) yet are fully consistent with a group of potential invertebrate prey including *D. villosus*, *P. antennarius*, and the dragonfly nymphs *Erithromma* sp. and *Ischnura* sp. *L. gibbosus* is known to have paleomonids, Odonata larval stages, and *D. villosus* as common items in its diet, yet it is also recognized as an active predator of the zebra mussel *D. polymorpha* [95,96,97,98,99]. Here, isotopic values and niche position suggest that the invasive bivalve does not contribute significantly to the diet of *L. gibbosus*, at least in winter. It is worth noting that the specimens of *L. gibbosus* analyzed in this study had a relatively small size, with a standard length ranging between 45 and 95 mm (Appendix A, Table A1). Mollusks become a significant component of the diet of the pumpkinseed only for individuals larger than 80–90 mm, e.g., [99]; thus, it is plausible that the pattern observed here might testify to a size-related dietary shift associated with a number of additional factors linked to, e.g., ontogeny or prey availability.

*Carassius auratus* showed a relatively high and diffuse overlap with a number of native species, such as *S. erythrophthalmus* and *S. squalus*, and to a lower extent with *A. anguilla*, all known to feed mainly on benthic invertebrates [100,101,102]. *C. auratus* has zooplanktivorous habits yet is able to shift to a benthivorous diet depending on resource availability [103,104,105]. In Lake Trasimeno, given the low abundance of zooplankton in winter months [29,106], it is likely that *C. auratus* modified its trophic habits, converging towards those characterizing native benthivorous species. A similar trophic shift may have occurred also for *Atherina boyeri*, showing a diffuse overlap with *S. squalus*, *S. erythrophthalmus*, *C. auratus*, and other benthivorous predators such as *C. carpio* and *T. tinca* [107,108]. Freshwater populations of *A. boyeri* are known to feed mainly on zooplankton, yet depending on size, prey availability, and local conditions, an important component of their diet in freshwater and transitional environments may be represented by benthic invertebrates such as amphipods and chironomids [109,110] see also [111,112] for confirmative examples from transitional environments.

Noticeably, *E. cisalpinus*, the only native piscivorous predator occurring in Lake Trasimeno, showed a negligible overlap with other introduced predators such as *P. fluviatilis* and *M. salmoides*, both showing significantly higher δ^15^N values than *E. cisalpinus*. In contrast, a relatively high overlap was observed between *E. cisalpinus* and *A. melas*. In winter months, the benthivorous *A. melas* may shift to piscivory, e.g., [113], while Crustacea may become a significant component of the diet of pikes, in particular for small-sized specimens [114,115,116]. Thus, given the relatively small size of *E. cisalpinus* individuals analyzed in the present study (222–412 mm standard length range; Appendix A, Table A1), it can be hypothesized that its diet converged towards that of *A. melas*, resulting in the observed high overlap.

## 5. Conclusions

The present study was carried out in winter, and additional analyses may provide a more advanced resolution of species interactions as affected by seasonal variation in, e.g., abundance and trophic habits. In addition, the location where invertebrate and fish consumers were sampled can be considered representative of the littoral benthic environments of the southern sectors of Lake Trasimeno [45]. However, given the 53 km long coastline of the basin [43], future analyses should include multiple locations to account for, e.g., local variations in the availability of basal resources such as primary producers [117,118] and how these influence the isotopic niches of consumers. Nonetheless, this investigation provided a first assessment of the potential for competition between non-indigenous and native invertebrates and fish currently occurring in Lake Trasimeno, indicating that isotopic approaches may represent a powerful tool for disentangling the complexity of the trophic interactions characterizing the food web of the lake, providing at the same time useful information for future actions of management and mitigation of the impact of non-indigenous species.

## Figures and Tables

**Table 1:** Non-indigenous invertebrate and fish species currently occurring in Lake Trasimeno. Information on the years of first record were collated from [24,53,54,55,56].

Group	Species	Year of First Record
Invertebrates	*Branchiura sowerbyi* Beddard 1892	<2000
	*Dikerogammarus villosus* Sowinsky 1894	2017
	*Dreissena polymorpha* Pallas 1771	1999
	*Physella acuta* Draparnaud, 1805 #	<1900
	*Procambarus clarkii* Girard 1852	2000
	*Sinanodonta woodiana* Lea 1834	2017
Fish	*Alburnus arborella* Bonaparte 1841	1975
	*Ameiurus melas* Rafinesque 1820	1984
	*Atherina boyeri* Risso 1810	1920
	*Carassius auratus* Linneus 1758	1990
	*Ctenopharyngodon idella* Valenciennes 1844 *	1986
	*Cyprinus carpio* Linneus 1758 #	Roman age
	*Gambusia holbrooki* Girard 1859	1927
	*Knipowitschia panizzae* Verga 1841	1976
	*Lepomis gibbosus* Linneus 1758	1926
	*Micropterus salmoides* Lacépède 1802	1990
	*Perca fluviatilis* Linneus 1758	1911
	*Pomatoschistus canestrinii* Ninni 1883	1988
	*Pseudorasbora parva* Temminck and Schlegels 1825	1999
	*Sabanejewia larvata* De Filippi 1859	1970
	*Silurus glanis* Linneus 1758 *	Unconfirmed

* Nonreproductive population. # Given the time since introduction, here, the species was considered autochthonous.

**Table 2:** SEAc and SEA_B_ of invertebrate consumers expressed as ‰^2^. For SEA_B_, modal values and the corresponding 95% confidence intervals (in italics) are included.

Taxon	SEAc	SEA_B_	95%	
*Anodonta anatina*	0.28	0.26	*0.09*	*0.49*
*Asellus aquaticus*	0.74	1.04	*0.44*	*1.56*
*Branchiura sowerbyi*	0.36	0.35	*0.19*	*0.67*
*Chironomus plumosus*	0.08	0.08	*0.03*	*0.16*
*Dikerogammarus villosus*	2.79	2.83	*2.09*	*3.63*
*Dreissena polymorpha*	0.23	0.23	*0.09*	*0.42*
*Echinogammarus veneris*	4.93	4.49	*2.91*	*7.26*
*Erpobdella octoculata*	0.2	0.21	*0.06*	*0.39*
*Erythromma* sp.	0.36	0.35	*0.17*	*0.58*
*Ischnura* sp.	0.07	0.08	*0.04*	*0.22*
*Palaemonetes antennarius*	1.72	1.56	*0.56*	*3.02*
*Physella acuta*	4.55	4.69	*1.48*	*8.03*
*Procambarus clarkii*	16.78	16.93	*9.68*	*25.28*
*Zooplankton*	1.12	1.09	*0.27*	*2.08*

**Table 3:** SEAc and SEA_B_ of fish consumers expressed as ‰^2^. For SEA_B_, modal values and the corresponding 95% confidence intervals (in italics) are included.

Taxon	SEAc	SEA_B_	95%	
*Ameiurus melas*	0.22	0.27	*0.15*	*0.38*
*Anguilla anguilla*	4.92	3.84	*1.65*	*5.94*
*Atherina boyeri*	1.29	1.18	*0.74*	*1.87*
*Carassius auratus*	2.12	1.91	*1.16*	*2.39*
*Cyprinus carpio*	0.29	0.24	*0.11*	*0.65*
*Esox cisalpinus*	0.02	0.02	*0*	*0.03*
*Lepomis gibbosus*	3.98	3.77	*2.47*	*5.65*
*Micropterus salmoides*	0.93	0.82	*0.37*	*2.01*
*Perca fluviatilis*	2.23	2.02	*1.28*	*3.25*
*Scardinius erythrophthalmus*	0.79	0.62	*0.29*	*1.38*
*Squalius squalus*	1.57	1.35	*0.93*	*2.17*
*Tinca tinca*	4.87	4.15	*2.12*	*6.25*

## Data Availability

Isotopic data used in this study are available upon request from the corresponding author. The data are not publicly available due to ongoing comparative analyses.

## Appendix A

**Table A1:** List of invertebrate and fish consumers sampled in the present study and in Mancini et al. [58]. The number of collected specimens is reported; for each taxon, the mean C:N ratio is included (±1 SE), together with the C:N ratio range, the mean length of individuals in mm (±1 SE), and the individual length range in mm. * = Shell length of bivalves.

Taxon	2018	2016	Total	Mean C:N	C:N Range	Mean Length	Length Range
Invertebrates							
*Anodonta anatina **	---	5	5	7.66 ± 0.61	7.04–8.32	99.7 ± 13.88	87–116
*Asellus aquaticus*	5	---	5	3.99 ± 1.12	2.77–5.78	8.47 ± 2.06	6.7–11.8
*Branchiura sowerbyi*	3	3	6	5.53 ± 0.23	5.22–5.75	46.83 ± 19.05	29–77
*Chironomus plumosus*	3	3	6	6.58 ± 0.39	6.14–7.09	5.8 ± 1.89	3.3–8.2
*Dikerogammarus villosus*	54	---	54	4.96 ± 0.45	4.31–5.9	12.71 ± 4.64	5.4–20.2
*Dreissena polymorpha **	---	8	8	4.65 ± 0.36	4.28–5.21	17.63 ± 5.93	10–26
*Echinogammarus veneris*	16	4	20	5.52 ± 0.9	4.39–8.03	7.37 ± 1.14	5.3–9.3
*Erpobdella octoculata*	3	3	6	4.25 ± 0.24	3.89–4.51	14.17 ± 4.49	7–19
*Erythromma* sp.	12	---	12	3.94 ± 0.17	3.66–4.18	14.52 ± 1.59	12.4–16.5
*Ischnura* sp.	6	---	6	3.74 ± 0.09	3.61–3.86	9.11 ± 1.34	7.5–11.5
*Palaemonetes antennarius*	4	2	6	3.85 ± 0.15	3.67–4.08	17.33 ± 3.88	12–22
*Physella acuta **	6	---	6	4.41 ± 0.33	4.04–4.87	9.5 ± 3.08	6–14
*Procambarus clarkii*	12	6	18	3.18 ± 0.25	2.33–3.46	97.26 ± 24.84	15–125
Zooplankton	---	5	5	7.49 ± 0.32	7.23–8.01	---	---
Fish							
*Ameiurus melas*	6	---	6	3.15 ± 0.05	3.09–3.24	188.75 ± 6.18	184–197
*Anguilla anguilla*	6	---	6	4.89 ± 1.92	3.75–8.44	401.25 ± 40.49	370–460
*Atherina boyeri*	21	---	21	3.13 ± 0.32	2.45–3.65	62.95 ± 13.95	35–80
*Carassius auratus*	15	---	15	3.16 ± 0.13	2.94–3.39	203.87 ± 94.52	91–312
*Cyprinus carpio*	6	---	6	3.2 ± 0.2	2.93–3.43	326.83 ± 100.75	240–480
*Esox cisalpinus*	8	---	8	3.12 ± 0.02	3.07–3.14	308.38 ± 74.41	222–412
*Lepomis gibbosus*	26	---	26	3.3 ± 0.05	3.21–3.45	76.5 ± 13.37	45–95
*Micropterus salmoides*	7	---	7	3.14 ± 0.1	3.06–3.29	183.86 ± 17.14	165–210
*Perca fluviatilis*	19	---	19	3.19 ± 0.08	3.03–3.33	145.16 ± 32.06	58–205
*Scardinius erythrophthalmus*	8	---	8	3.11 ± 0.1	2.96–3.24	143.43 ± 22.45	120–182
*Squalius squalus*	8	---	8	3.29 ± 0.12	3.04–3.48	99.67 ± 46.65	53–150
*Tinca tinca*	10	---	10	3.23 ± 0.1	3.18–3.46	243.25 ± 45.96	160–300

## Appendix B

**Table A2:** PERMANOVA post hoc bivariate comparisons performed on δ^13^C and δ^15^N values of invertebrate consumers. Monte Carlo-corrected probability values (*P_Monte Carlo_*) are reported. Significant *P_Monte Carlo_* values are indicated in bold. Codes in parentheses refer to those reported in column headings.

Taxon	1	2	3	4	5	6	7	8	9	10	11	12	13
*Anodonta anatina* (1)													
*Asellus aquaticus* (2)	**0.0002**												
*Branchiura sowerbyi* (3)	**0.02**	**0.0009**											
*Chironomus plumosus* (4)	**0.0003**	**0.0003**	0.13										
*Dikerogammarus villosus* (5)	**0.0001**	**0.0001**	**0.0001**	**0.0001**									
*Dreissena polymorpha* (6)	**0.0001**	**0.0001**	**0.0001**	**0.0001**	**0.0001**								
*Echinogammarus veneris* (7)	**0.01**	**0.0004**	**0.03**	0.12	**0.0001**	**0.0001**							
*Erpobdella octoculata* (8)	**0.0001**	**0.0001**	**0.0001**	**0.0001**	**0.0001**	**0.0001**	**0.0007**						
*Erythromma* sp. (9)	**0.0001**	**0.0001**	**0.0001**	**0.0001**	0.09	**0.0001**	**0.0001**	**0.0001**					
*Ischnura* sp. (10)	**0.0001**	**0.0002**	**0.0001**	**0.0001**	0.08	**0.0001**	**0.0004**	**0.0001**	0.45				
*Palaemonetes antennarius* (11)	**0.0001**	**0.0001**	**0.0001**	**0.0001**	**0.002**	**0.0001**	**0.0001**	**0.0001**	**0.0001**	**0.0001**			
*Physella acuta* (12)	0.15	**0.001**	**0.02**	**0.02**	**0.0001**	**0.0001**	**0.02**	**0.0013**	**0.0001**	**0.0001**	**0.0001**		
*Procambarus clarkii* (13)	0.17	0.07	0.31	0.49	**0.0001**	**0.0001**	0.51	**0.03**	0.07	0.19	**0.001**	0.12	
Zooplankton (14)	0.49	**0.002**	0.44	0.17	**0.0001**	**0.0001**	**0.05**	**0.0001**	**0.0001**	**0.0001**	**0.0001**	0.18	0.29

**Table A3:** PERMANOVA post hoc bivariate comparisons performed on δ^13^C and δ^15^N values of fish consumers. Monte Carlo-corrected probability values (*P_Monte Carlo_*) are reported. Significant *P_Monte Carlo_* values are indicated in bold. Codes in parentheses refer to those reported in column headings.

Taxon	1	2	3	4	5	6	7	8	9	10	11
*Ameiurus melas* (1)											
*Anguilla anguilla* (2)	**0.002**										
*Atherina boyeri* (3)	**0.001**	**0.001**									
*Carassius auratus* (4)	**0.001**	0.35	**0.002**								
*Cyprinus carpio* (5)	**0.001**	**0.02**	**0.005**	0.03							
*Esox cisalpinus* (6)	0.94	**0.001**	**0.001**	**0.001**	**0.001**						
*Lepomis gibbosus* (7)	**0.001**	**0.001**	**0.001**	**0.001**	**0.001**	**0.001**					
*Micropterus salmoides* (8)	**0.01**	**0.001**	**0.001**	**0.001**	**0.001**	**0.003**	**0.001**				
*Perca fluviatilis* (9)	**0.05**	**0.001**	**0.001**	**0.001**	**0.001**	**0.05**	**0.001**	**0.04**			
*Scardinius erythrophthalmus* (10)	**0.001**	0.25	**0.002**	0.91	**0.02**	**0.001**	**0.001**	**0.001**	**0.001**		
*Squalius squalus* (11)	**0.001**	**0.04**	**0.002**	0.11	0.47	**0.001**	**0.001**	**0.001**	**0.001**	0.17	
*Tinca tinca* (12)	**0.001**	0.43	**0.001**	0.007	**0.002**	**0.001**	**0.001**	**0.001**	**0.001**	**0.01**	**0.002**

## Appendix C

**Table A4:** Interspecific overlaps in SEA_c_ and modal SEA_B_ values of non-indigenous and native invertebrate taxa. For the sake of succinctness, values lower than 0.01‰^2^ are reported as “<0.01”. Percent overlaps are reported for both metrics; those lower than 1% are reported as “<1%”. For SEA_B_ modal values, 95% confidence intervals are included in italics.

NIS	Native	Overlap (SEA_c_)	% Overlap (SEA_c_)	Overlap (SEA_B_)	% Overlap (SEA_B_)	95% CI	
*Branchiura sowerbyi*	*Chironomus p* *lumosus*	<0.01	<1%	<0.01	<1%	*<1%*	*<1%*
	*Echinogammarus veneris*	0.07	1.36	0.08	1.37	*1.19*	*1.54*
	*Physella acuta*	0.13	2.75	0.08	1.96	*<1%*	*4.35*
*Dikerogammarus villosus*	*Erythromma* sp.	0.32	11.36	0.39	9.44	*9.43*	*9.46*
	*Ischnura* sp.	0.07	2.66	0.11	2.87	*2.62*	*2.93*
*Dreissena polymorpha*	*Anodonta anatina*	<0.01	<1%	<0.01	<1%	*<1%*	*<1%*
	*Zooplankton*	<0.01	<1%	<0.01	<1%	*<1%*	*<1%*
*Procambarus clarkii*	*Asellus aquaticus*	<0.01	<1%	<0.01	<1%	*<1%*	*<1%*
	*Echinogammarus veneris*	3.02	16.15	4.82	17.94	*11.99*	*24.58*
	*Erpobdella octoculata*	<0.01	<1%	<0.01	<1%	*<1%*	*<1%*
	*Erythromma* sp.	0.36	2.12	0.44	1.77	*1.74*	*1.81*
	*Ischnura* sp.	0.07	<1%	0.13	<1%	*<1%*	*<1%*
	*Physella acuta*	1.35	6.74	2.26	8.45	*8.34*	*8.57*

**Table A5:** Interspecific overlaps in SEA_c_ and modal SEA_B_ values of native invertebrate taxa. For the sake of succinctness, values lower than 0.01‰^2^ are reported as “<0.01”. Percent overlaps are reported for both metrics; those lower than 1% are reported as “<1%”. For SEA_B_ modal values, 95% confidence intervals are included in italics.

Native	Native	Overlap (SEA_c_)	% Overlap (SEA_c_)	Overlap (SEA_B_)	% Overlap (SEA_B_)	95% CI	
*Echinogammarus veneris*	*Asellus aquaticus*	<0.01	<1%	<0.01	<1%	*<1%*	*<1%*
	*Chironomus plumosus*	<0.01	<1%	<0.01	<1%	*<1%*	*<1%*
*Erpobdella octoculata*	*Asellus aquaticus*	<0.01	<1%	<0.01	<1%	*<1%*	*<1%*
	*Echinogammarus veneris*	<0.01	<1%	<0.01	<1%	*<1%*	*<1%*
*Ischnura* sp.	*Erythromma* sp.	0.02	5.79	0.03	6.02	*3.31*	*8.45*
*Physella acuta*	*Chironomus plumosus*	0.08	1.86	0.07	1.15	*0.05*	*3.49*
	*Echinogammarus veneris*	2.47	35.21	3.16	30.95	*22.84*	*42.12*
*Zooplankton*	*Anodonta anatina*	0.24	20.38	0.27	18.12	*10.22*	*26.04*
	*Chironomus plumosus*	0.05	3.88	0.05	4.01	*2.33*	*5.86*
	*Physella acuta*	0.22	4	0.14	3.74	*2.72*	*5.15*

**Table A6:** Interspecific overlaps in SEAc and modal SEAB values of non-indigenous invertebrate taxa. For the sake of succinctness, values lower than 0.01‰2 are reported as “<0.01”. Percent overlaps are reported for both metrics; those lower than 1% are reported as “<1%”. For SEAB modal values, 95% confidence intervals are included in italics.

NIS	NIS	Overlap (SEA_c_)	% Overlap (SEA_c_)	Overlap (SEA_B_)	% Overlap (SEA_B_)	95% CI	
*Procambarus clarkii*	*Branchiura sowerbyi*	<0.01	<1%	<0.01	<1%	*<1%*	*<1%*
	*Dikerogammarus villosus*	2.73	16.22	3.59	15.83	*10.38*	*19.28*

## Appendix D

**Table A7:** Interspecific overlaps in SEA_c_ and modal SEA_B_ values of non-indigenous and native fish taxa. For the sake of succinctness, values lower than 0.01‰^2^ are reported as “<0.01”. Percent overlaps are reported for both metrics; those lower than 1% are reported as “<1%”. For SEA_B_ modal values, 95% confidence intervals are included in italics.

NIS	Native	Overlap (SEA_c_)	% Overlap (SEA_c_)	Overlap (SEA_B_)	% Overlap (SEA_B_)	95% CI	
*Ameiurus melas*	*Anguilla anguilla*	<0.01	<1%	<0.01	<1%	*<1%*	*<1%*
	*Cyprinus carpio*	<0.01	<1%	<0.01	<1%	*<1%*	*<1%*
	*Esox cisalpinus*	<0.01	2.73	<0.01	2.32	*1.54*	*3.03*
	*Scardinius erythrophthalmus*	<0.01	<1%	<0.01	<1%	*<1%*	*<1%*
	*Squalius squalus*	<0.01	<1%	<0.01	<1%	*<1%*	*<1%*
*Atherina boyeri*	*Anguilla anguilla*	0.53	9.24	0.89	10.44	*8.09*	*12.81*
	*Cyprinus carpio*	<0.01	<1%	<0.01	<1%	*<1%*	*<1%*
	*Esox cisalpinus*	<0.01	<1%	<0.01	<1%	*<1%*	*<1%*
	*Scardinius erythrophthalmus*	<0.01	<1%	<0.01	<1%	*<1%*	*<1%*
	*Squalius squalus*	0.34	13.28	0.29	12.59	*7.82*	*14.52*
*Carassius auratus*	*Anguilla anguilla*	1.4	24.79	1.67	23.7	*21.27*	*26.23*
	*Cyprinus carpio*	0.04	1.69	0.05	2.21	*1.06*	*3.51*
	*Esox cisalpinus*	<0.01	<1%	<0.01	<1%	*<1%*	*<1%*
	*Scardinius erythrophthalmus*	0.65	29.02	0.63	25.1	*22*	*32.17*
	*Squalius squalus*	0.62	20.32	0.75	18.46	*17.66*	*22.54*
	*Tinca tinca*	0.06	<1%	0.08	<1%	*<1%*	*1.31*
*Micropterus salmoides*	*Anguilla anguilla*	<0.01	<1%	<0.01	<1%	*<1%*	*<1%*
	*Cyprinus carpio*	<0.01	<1%	<0.01	<1%	*<1%*	*<1%*
	*Esox cisalpinus*	<0.01	<1%	<0.01	<1%	*<1%*	*<1%*
	*Scardinius erythrophthalmus*	<0.01	<1%	<0.01	<1%	*<1%*	*<1%*
	*Squalius squalus*	<0.01	<1%	<0.01	<1%	*<1%*	*<1%*
*Perca fluviatilis*	*Anguilla anguilla*	<0.01	<1%	<0.01	<1%	*<1%*	*<1%*
	*Cyprinus carpio*	<0.01	<1%	<0.01	<1%	*<1%*	*<1%*
	*Esox cisalpinus*	<0.01	<1%	<0.01	<1%	*<1%*	*<1%*
	*Scardinius erythrophthalmus*	<0.01	<1%	<0.01	<1%	*<1%*	*<1%*
	*Squalius squalus*	<0.01	<1%	<0.01	<1%	*<1%*	*<1%*
	*Tinca tinca*	<0.01	<1%	<0.01	<1%	*<1%*	*<1%*

**Table A8:** Interspecific overlaps in SEA_c_ and modal SEA_B_ values of native fish taxa. For the sake of succinctness, values lower than 0.01‰^2^ are reported as “<0.01”. Percent overlaps are reported for both metrics; those lower than 1% are reported as “<1%”. For SEA_B_ modal values, 95% confidence intervals are included in italics.

Native	Native	Overlap (SEA_c_)	% Overlap (SEA_c_)	Overlap (SEA_B_)	% Overlap (SEA_B_)	95% CI	
*Anguilla anguilla*	*Cyprinus carpio*	0.29	5.89	0.31	5.05	*3.56*	*6.36*
	*Esox cisalpinus*	<0.01	<1%	<0.01	<1%	*<1%*	*<1%*
	*Scardinius erythrophthalmus*	0.77	15.54	0.69	10.71	*8.02*	*16.25*
	*Squalius squalus*	1.21	22.88	1.28	18.14	*16.07*	*23.24*
	*Tinca tinca*	1.04	11.85	0.97	11.33	*8.12*	*12.38*
*Scardinius erythrophthalmus*	*Esox lucius*	<0.01	<1%	<0.01	<1%	*<1%*	*<1%*
	*Cyprinus carpio*	0.02	1.72	<0.01	1.6	*1.25*	*1.93*
*Squalius squalus*	*Esox lucius*	<0.01	<1%	<0.01	<1%	*<1%*	*<1%*
	*Cyprinus carpio*	0.15	8.66	0.08	7.21	*5.53*	*9.43*
	*Scardinius erythrophthalmus*	0.31	15.12	0.37	12.62	*11.72*	*14.89*
*Tinca tinca*	*Scardinius erythrophthalmus*	0.04	<1%	0.07	1.05	*<1%*	*1.16*

**Table A9:** Interspecific overlaps in SEA_c_ and modal SEA_B_ values of non-indigenous fish taxa. For the sake of succinctness, values lower than 0.01‰^2^ are reported as “<0.01”. Percent overlaps are reported for both metrics; those lower than 1% are reported as “<1%”. For SEA_B_ modal values, 95% confidence intervals are included in italics.

NIS	NIS	Overlap (SEA_c_)	% Overlap (SEA_c_)	Overlap (SEA_B_)	% Overlap (SEA_B_)	95% CI	
*Atherina boyeri*	*Ameiurus melas*	<0.01	<1%	<0.01	<1%	*<1%*	*<1%*
*Carassius auratus*	*Ameiurus melas*	<0.01	<1%	<0.01	<1%	*<1%*	*<1%*
	*Atherina boyeri*	0.39	13.1	0.61	18.77	*12.02*	*20.58*
*Micropterus salmoides*	*Ameiurus melas*	<0.01	<1%	<0.01	<1%	*<1%*	*<1%*
	*Atherina boyeri*	<0.01	<1%	<0.01	<1%	*<1%*	*<1%*
	*Carassius auratus*	<0.01	<1%	<0.01	<1%	*<1%*	*<1%*
*Perca fluviatilis*	*Ameiurus melas*	0.13	5.56	0.27	7.59	*4.54*	*7.66*
	*Micropterus salmoides*	0.29	10.26	0.34	10.69	*8.09*	*12.33*
	*Atherina boyeri*	<0.01	<1%	<0.01	<1%	*<1%*	*<1%*
	*Carassius auratus*	<0.01	<1%	<0.01	<1%	*<1%*	*<1%*
